# SERS Barcode Libraries: A Microfluidic Approach

**DOI:** 10.1002/advs.201903172

**Published:** 2020-04-22

**Authors:** Semih Sevim, Carlos Franco, Xiang‐Zhong Chen, Alessandro Sorrenti, David Rodríguez‐San‐Miguel, Salvador Pané, Andrew J. deMello, Josep Puigmartí‐Luis

**Affiliations:** ^1^ Institute of Chemical and Bioengineering ETH Zurich Vladimir Prelog Weg 1 Zurich 8093 Switzerland; ^2^ Multi‐Scale Robotics Lab (MSRL) Institute of Robotics and Intelligent Systems ETH Zurich Tannenstrasse 3 Zurich 8092 Switzerland

**Keywords:** microengineered SERS substrates, microfluidics, multiple detection, SERS barcoding

## Abstract

Microfluidic technologies have emerged as advanced tools for surface‐enhanced Raman spectroscopy (SERS). They have proved to be particularly appealing for in situ and real‐time detection of analytes at extremely low concentrations and down to the 10 × 10^−15^ m level. However, the ability to prepare reconfigurable and reusable devices endowing multiple detection capabilities is an unresolved challenge. Herein, a microfluidic‐based method that allows an extraordinary spatial control over the localization of multiple active SERS substrates in a single microfluidic channel is presented. It is shown that this technology provides for exquisite control over analyte transport to specific detection points, while avoiding cross‐contamination; a feature that enables the simultaneous detection of multiple analytes within the same microfluidic channel. Additionally, it is demonstrated that the SERS substrates can be rationally designed in a straightforward manner and that they allow for the detection of single molecules (at concentrations as low as 10^−14^ m). Finally, it is shown that rapid etching and reconstruction of SERS substrates provides for reconfigurable and reusable operation.

## Graphical abstract

A microfluidic approach for surface‐enhanced Raman spectroscopy (SERS) substrate fabrication and multiple analyte detection is presented. Combining the controlled diffusion of analytes with pneumatic clamp actuation enables the spatially controlled synthesis of custom shaped SERS substrates, where regioselective localization and detection is ensured. The method enables the reconfiguration and reuse of these substrates without cross‐contamination, hence generating SERS barcodes.

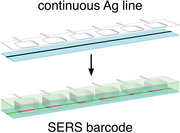

Ever since the discovery of the surface‐enhanced Raman scattering (SERS),^[^ ^1^ ^]^ much attention has focused on the preparation of metallic‐based nanostructures in a facile, robust, and rapid manner.^[^ ^2^, ^3^ ^]^ In this respect, the controlled chemical reduction of noble metals is considered the gold‐standard approach in generating SERS substrates enabling analyte detection at concentration down to 10^−13^ m.^[^ ^4^, ^5^ ^]^ Recently, the combination of such synthetic approaches with the use of compliant support layers has been used to obtain transparent, flexible, and highly active SERS substrates.^[^ ^6^, ^7^, ^8^ ^]^ However, new advances in this area rely on the controlled positioning of SERS substrates onto the support layer and the controlled diffusion of probe molecules (analytes) to SERS active sites for their in situ and real‐time detection.^[^ ^9^ ^]^

Accordingly, top‐down technologies based on microfluidic principles have emerged as advanced tools in several SERS studies and applications.^[^ ^10^ ^]^ Microfluidic devices provide a suitable environment for the generation of SERS arrays at predefined locations inside a microfluidic channel, e.g., via laser‐induced photoreduction.^[^ ^4^, ^11^, ^12^, ^13^ ^]^ In addition, microfluidic methods have the potential for an exquisite control over the diffusion of analytes to specific detection points.^[^ ^14^ ^]^ Nonetheless, note that microfluidic‐based SERS approaches are still at their infancy, and many challenges have not been addressed yet.^[^ ^9^ ^]^ Such challenges include the facile preparation of SERS substrates as reconfigurable and reusable devices, the controllable trapping and release of probe molecules without cross‐contamination, and the ability to detect multiple species within a single microfluidic channel.

Herein, we present a new microfluidic‐based method that addresses these issues enabling multiple SERS substrates to be produced in a single step inside a single microfluidic channel. The method is cost‐effective, reproducible, highly sensitive, and enables detection at concentrations as low as 10^−14^ m. Additionally, the SERS substrates can be synthetized with bespoke aspect ratios combining the controlled diffusion of reagent‐laden flows and pneumatic clamp actuation (vide infra). Specifically, we demonstrate that these two microfluidic features allow: i) to control the spatial localization of SERS substrates as well as analytes at specific sites along a single microfluidic channel, ii) to prevent cross‐contamination, and iii) to achieve multiple detection capabilities, thus resulting in SERS barcodes. Furthermore, our approach enables the etching and reconstruction of SERS substrates in a rapid and continuous fashion, thus facilitating the fabrication of reconfigurable and reusable SERS platforms. It should be noted that, even if the potential of our methodology is exemplified here with the formation of SERS barcodes, it is a general procedure that can be extended to accomplish a precise localization of other functional materials and/or molecules.

Double‐layer microfluidic devices were used for all the experiments. Briefly, these devices consist of two polydimethylsiloxane (PDMS) layers bonded together, i.e., a control layer (top layer) and a fluidic layer (bottom layer) (**Figure** **1**a). It is important to note that, a thin PDMS membrane is present between the control layer and the fluidic layer (see the Experimental Section and the Supporting Information for fabrication details). While the fluidic layer, which is bonded to a glass surface, is used to flow reagent‐laden solutions (Figure 1b(i)), the control layer is used to partially squeeze the membrane toward the glass substrate (Figure 1b(ii)). Deformation of the membrane, and hence of the fluidic layer, is only achieved when nitrogen gas is injected into the control layer.^[^ ^15^ ^]^ Figure 1b illustrates the principle of our method. The controlled pneumatic actuation of consecutive squared pneumatic clamps allows to precisely localize multiple distinct specimens (i.e., SERS substrates or analytes) on the surface of the same microfluidic channel (Figure 1b(i–iv)). For example, Figure 1b shows the localization and positioning of three different specimens (blue, red, and green) by sequential trapping in a single channel. Note that the sequential localization of specimens requires additional washing steps after each sample deposition to eliminate surplus solutions, while keeping the desired specimens in place through applying a sufficient pressure to the pneumatic clamps.

**Figure 1:**
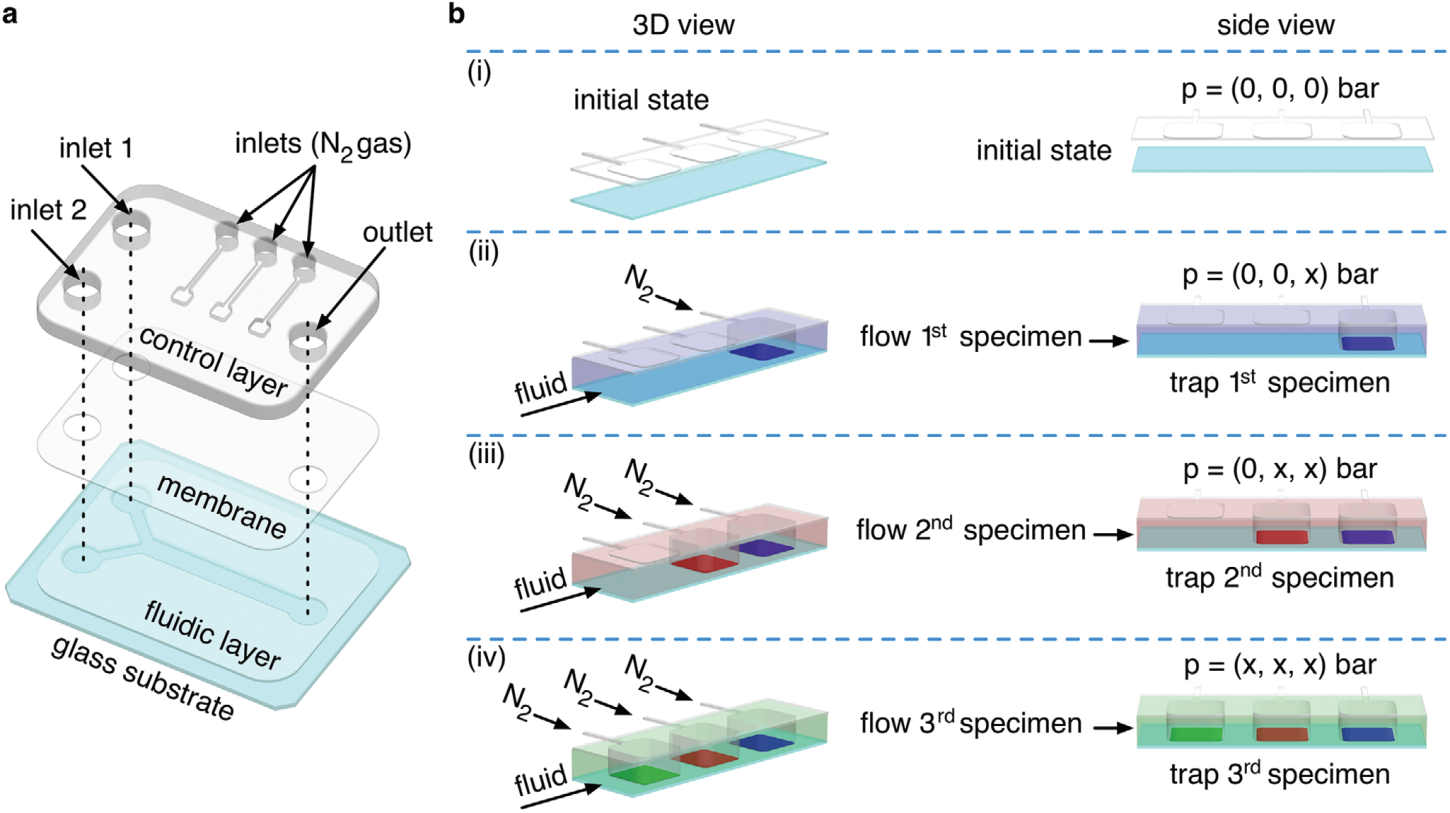
a) Exploded drawing of the double‐layer microfluidic device used in experiments. b) Illustrations showing the controlled localization of three specimens (blue, red, and green) within the fluidic layer of the microfluidic device shown in (a). Panels (i–iv) show the sequence of steps followed to trap the three specimens. The “x” indicates pneumatic actuation with nitrogen gas.

We initially studied the electroless deposition of silver (Ag) inside our double‐layer microfluidic devices. Ag was chosen as a SERS substrate, since it provides a greater enhancement of SERS signals when compared to other noble metals.^[^ ^9^ ^]^ The electroless deposition of Ag was performed via free diffusion of Ag salt (AgX) and reducing agent (Red) solutions inside the fluidic layer (see the Supporting Information for details). For simplicity, in these initial experiments, the fluidic layer comprised only a single inlet and an outlet (Figure S1a, Supporting Information). After injection of AgX and Red solutions inside the fluidic layer, the formation of an Ag film was observed within 5 min (see Figure S1b, Supporting Information). Scanning electron microscopy (SEM) and energy dispersive X‐ray (EDX) measurements indicated that the deposited Ag was primarily composed of clustered Ag nanoparticles, with an average particle size of 100 ± 25 nm (Figure S2a,b, Supporting Information). Moreover, X‐ray diffraction (XRD) analysis of the Ag film indicated the formation of Ag with a face‐centered cubic (fcc) structure (Figure S2c, Supporting Information).^[^ ^16^ ^]^ Additionally, pressurization with nitrogen gas (3 bar) of one of the square pneumatic clamps located in the control layer (Figure 1a) could clearly prevent the electroless deposition of Ag underneath the clamp (Figure S1c, Supporting Information). In other words, the partial deformation (or partial squeezing) of the membrane toward the glass substrate led to the fabrication of Ag films with a microengineered empty patch, with the same shape as the pneumatic clamp. Additionally, to ensure that electroless deposition of Ag does not occur in the empty patch prior to the characterization of the Ag film, a washing step with deionized (DI) water was performed to eliminate unreacted surplus solutions while keeping the pneumatic clamp actuated.

To increase the number of microengineered Ag structures prepared by electroless deposition, we decided to add a second inlet into our double‐layer microfluidic device (inlet 2 in Figure 1a) and study the formation of the Ag structures under continuous flow. In this case, AgX and Red solutions were injected into the fluidic layer from inlet 1 and inlet 2, respectively (Figure 1a and the Supporting Information for further details). We observed that when equal flow rates were used, an Ag line was patterned at the center of the main microfluidic channel on the glass substrate. We refer to this process as to in‐flow Ag patterning.^[^ ^17^ ^]^ Additionally, we showed that a total flow rate (TFR) of 100 µL min^−1^ generates Ag lines that are ≈10 µm wide, while a TFR of 20 µL min^−1^ leads to 40 µm wide lines (Figure S3b,c, Supporting Information). These results clearly evidence that Ag lines with different aspect ratios can be deposited in a straightforward manner employing a Y‐shape fluidic layer.

Despite the fact that Ag lines and films could be rapidly generated using our approach (Figures S3b,c and S1b, respectively, Supporting Information), an additional etching step after the in‐flow Ag patterning process was needed to generate multiple microengineered Ag structures within a single microfluidic channel. Accordingly, we investigated the controlled etching of Ag achieved by flowing throughout the fluidic layer a saturated solution of 7,7,8,8‐tetracyanoquinodimethane (TCNQ) in acetonitrile. TCNQ is a well‐known oxidant for Ag.^[^ ^18^ ^]^ As shown in Movie S1 (Supporting Information), a 300 µm wide Ag film could be completely removed in about 15 min from the microfluidic channel by injecting the TCNQ solution at TFR of 50 µL min^−1^. Consequently, combining the pneumatic actuation of a single clamp with the controlled chemical reduction and oxidation of Ag, we could produce one or two segmented Ag lines as well as an Ag film (taking the shape of the pneumatic clamp) with an excellent spatial control inside the fluidic layer (**Figure** **2**a–c). Note that, the patterning of two segmented Ag lines (Figure 2b) required dilution of the AgX and Red solutions (see the Supporting Information for further details) to avoid unwanted deposition of Ag between the lines, yielding undefined Ag structures. Additionally, increasing the number of pneumatic clamps in the control layer enabled the parallelized synthesis of various microengineered Ag structures in a single microfluidic channel (Figure 2d; and Figure S3d, Supporting Information). Furthermore, once formed, the localized and parallelized Ag structures could be selectively removed by injecting a saturated TCNQ solution in acetonitrile with the release of the corresponding pneumatic clamp (Figure 2d,e). This etching method facilitates the efficient cleaning and subsequent reuse of the microfluidic device. To better understand the mechanism undergoing the oxidation of the electroless deposited Ag, the pressure of the pneumatic clamp was reduced from 3 to 1 bar.^[^ ^15^ ^]^ Interestingly, we observed that lowering the pressure to 1 bar favored the controlled diffusion of TCNQ underneath the actuated membrane, which led to the oxidation of Ag and to the in situ formation of AgTCNQ, a well‐known semiconducting metal–organic charge transfer complex (Figure S4, Supporting Information).^[^ ^15^, ^19^, ^20^, ^21^ ^]^ Conversely, use of a 3 bar pressure prevented TCNQ diffusion below the membrane, and hence, the microengineered Ag structures could be preserved on the glass substrate (Figure 2e), even at TFR as high as 100 µm min^−1^. Remarkably, we noticed that the partial actuation of the pneumatic clamp (at 1 bar) enabled a controlled growth of AgTCNQ either forming a radial pattern or perfectly aligned wires when Ag films or Ag lines were used as a template for their growth, respectively (Figure S4, Supporting Information). The AgTCNQ wires obtained were tens of micrometer in length (29 µm ± 7 µm) and less than 3 µm in width. AgTCNQ wires were characterized using EDX and Raman spectroscopy, as well as electrical measurements. Significantly, all data clearly confirmed the formation of the AgTCNQ charge transfer complex (Figure S5a–c, Supporting Information). Notably, these results clearly indicate that pneumatic clamp actuation can be exploited to control the deposition of multiple crystalline functional materials at specific locations on surfaces due to the controlled diffusion of reagents underneath the actuated membrane.

**Figure 2:**
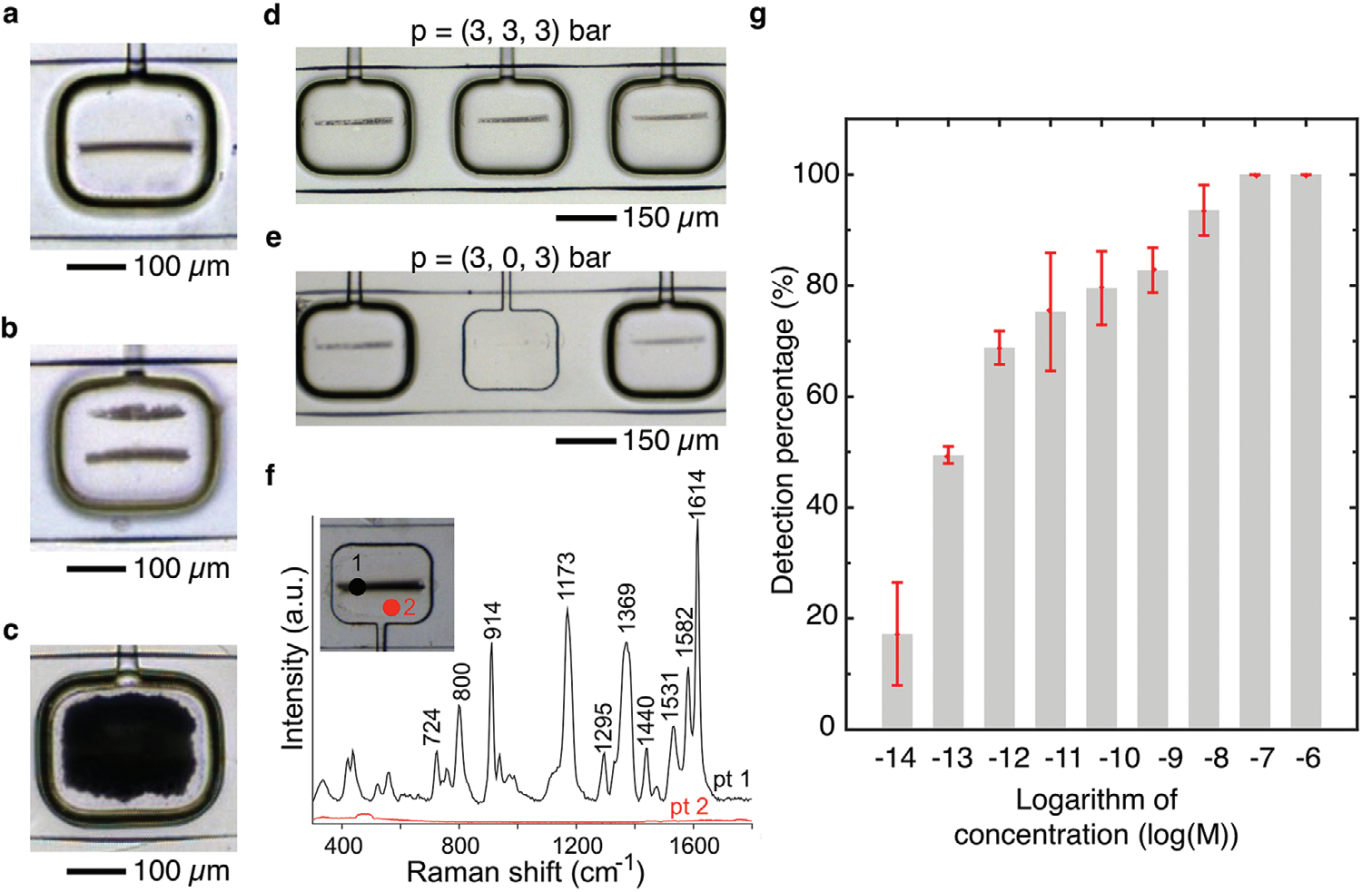
a–c) Microscope images of different Ag structures patterned under a pneumatic clamp: a) a segmented Ag line, b) two segmented Ag lines, and c) an Ag film. d) Microscope image showing three segmented Ag lines patterned under adjacent pneumatic clamps, and e) after a selective etching step. f) Raman spectra acquired on a segmented Ag line (point 1, black spectra), and on the glass slide (point 2, red spectra), when a 10^−6^ m crystal violet solution is injected in the fluidic layer. The inset micrograph indicates the two representative positions where each measurement was made. g) Variation of the detection event percentage as a function of CV solution concentration (between 10^−14^ m and 10^−6^ m).

Subsequently, we assessed the performance of the segmented Ag lines as SERS active substrates using crystal violet (CV) as a model Raman probe molecule. We chose CV because the Raman fingerprint for this molecule are extensively studied in SERS spectroscopy.^[^ ^13^, ^22^, ^23^ ^]^ In a typical experiment, an aqueous solution of CV (at 10^−6^ m) was injected over a period of 4 min at a constant TFR of 50 µL min^−1^. The flow was then stopped, and the Raman spectra acquired just afterward. As shown in Figure 2f, while no Raman signals were detected outside the segmented Ag lines (e.g., point 2 in Figure 2f), measurements performed on top of segmented Ag lines gave rise to intense Raman spectra (e.g., point 1 in Figure 2f). The Raman spectra show bands located at 724, 800, 914, 1173, 1295, 1369, 1440, 1531, 1582, and 1614 cm^−1^, which are characteristic for CV.^[^ ^13^, ^22^, ^23^ ^]^ The generality of our approach was also tested with a different Raman probe, namely 4‐aminothiophenol (4‐ATP) and a protein, namely bovine serum albumin, see Figures S6 and S7, respectively (Supporting Information). Consequently, these data indicate that microengineered Ag substrates act as effective “hot spots,” allowing detection of different analytes in solution.^[^ ^24^ ^]^

To assess the SERS performances of our Ag structures, we injected CV solutions at different concentrations ranging between 10^−14^ m and 10^−6^ m.^[^ ^4^, ^13^, ^25^, ^26^ ^]^ Each solution was injected following the same protocol described above (TFR of 50 µL min^−1^ for a period of 4 min). SERS measurements were conducted at 31 selected detection points along a presynthesized and washed Ag line, with each experiment being repeated three times for each CV concentration to conduct statistical analysis. Figure 2g shows that at CV concentration as low as 10^−13^ m, we observed a successful analyte detection in 50% of the measurements (see the Experimental Details in the Supporting Information), whereas CV was still detectable at 10^−14^ m in 20% of measurements (Figure S8, Supporting Information). In other words, at concentration < 10^−13^ m we are in a stochastic detection regime with an acceptable probability of detecting CV at concentrations as low as 10^−14^ m. Note that, a stochastic SERS detection observed at low concentrations is ascribed by several authors to single/a few molecule detection events.^[^ ^13^, ^26^, ^27^ ^]^

We note that, while CV could be detected at concentrations as low as 10^−14^ m under the above described conditions (stopped flow), real‐time monitoring of CV Raman signals under continuous flow of the analyte reached the stochastic detection regime at higher concentrations. Specifically, when flowing a CV solution at 50 µL min^−1^, the detection of CV Raman signals was found to be highly stochastic already at a concentration of 10^−9^ m (Figures S9 and S10, Supporting Information).

To clearly demonstrate that the pneumatic actuation can also prevent cross‐contamination, we explored the detection of different analytes using parallelized in‐flow patterned segmented Ag lines. Specifically, we selected three model Raman probe molecules, namely CV, 4‐ATP, and Rhodamine 6G (R6G). **Figure** **3**a shows a schematic illustration of the consecutive steps undertaken for the detection of three analytes in the same microfluidic channel. Initially, three segmented Ag lines were generated as discussed above (Figure 2d) and then, washed with DI water (washing step 1, Figure 3a(i)).^[^ ^28^ ^]^ Subsequently, a solution of CV (10^−6^ m) was flowed throughout the fluidic layer for 10 min (TFR = 50 µL min^−1^) to label the first segmented Ag line present in the microfluidic channel (Figure 3a(ii)). Note that, during this labeling step, the first pneumatic clamp was not actuated (0 bar), whereas the other two clamps were actuated at 3 bar to avoid the diffusion of CV to the Ag lines located underneath them (Figure 3a(ii)). Next, the main microfluidic channel was washed with DI water for 3 min to remove residual CV solution (washing step 2, Figure 3a(ii)).^[^ ^28^ ^]^ During this washing step, all the three pneumatic clamps were actuated at 3 bar to prevent washing away the analyte (vide infra **Figure** **4**a), as well as to avoid cross‐contamination. Finally, this sequence of steps was repeated to label the second and third segmented Ag lines with 4‐ATP (10^−6^ m) and R6G (10^−6^ m), respectively (Figure 3a(iii,iv)). Figure 3c shows the color‐coded Raman map of the segmented Ag lines shown in Figure 3b after CV, 4‐ATP, and R6G labeling is completed, with colors corresponding to characteristic Raman peaks of CV (914 cm^−1^), 4‐ATP (1074 cm^−1^), and R6G (1512 cm^−1^). The results obtained clearly confirm that our methodology relying on the pneumatic clamp actuation can enable multiple SERS detections inside a single microfluidic channel, while avoiding cross‐contamination. To further demonstrate the uniformity of the SERS signal along the Ag substrate and the complete absence of cross‐contamination, we performed a high‐resolution Raman mapping of two adjacent Ag lines labeled, respectively, with CV and 4‐ATP (Figure S11, Supporting Information).

**Figure 3:**
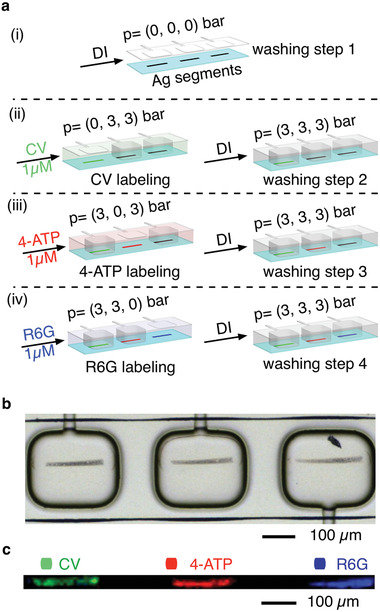
a) From (i–iv) schematic illustrations of the sequential steps followed to achieve the simultaneous detection of three model Raman probe molecules on adjacent segmented Ag lines located within a single microfluidic channel. b) Microscope image of the three segmented Ag lines used in the experiment. c) False color Raman map of the three model Raman probe molecules on each segmented Ag line shown in (b). CV is represented by green, 4‐ATP by red, and R6G by blue.

**Figure 4:**
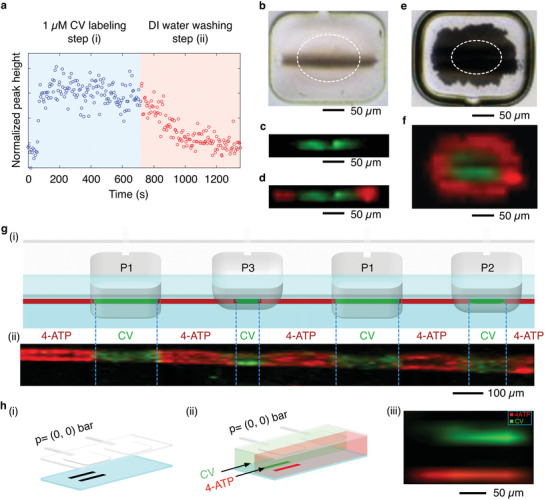
a) Variation of Raman intensity (at 914 cm^−1^) during the labeling of a segmented Ag line (step (i)) and the washing process (step (ii)). b) Optical microscope image of a partially actuated pneumatic clamp with a segmented Ag line underneath it. c,d) Raman maps of CV and a 4‐ATP/CV/4‐ATP sequence, respectively. e) Optical microscope image of an Ag film. f) Raman map of a 2D barcode. The dashed lines in (b,e) highlight the area of the membrane in contact with the glass substrate. g) Illustration of a 1D barcode in (i) and the corresponding false color Raman map in (ii). h) From (i–ii), schematic illustrations of a simultaneous detection experiment, and in (iii) the corresponding false color Raman map. In all Raman maps the characteristic peaks used during the measurements were: 914 cm^−1^ for CV (green) and 1074 cm^−1^ for 4‐ATP (red). Note that the clamp actuation was not required during the experiment in (h).

To go a step further, we investigated the potential of our methodology for labeling a single Ag substrate with two different analytes delivered at specific positions. Figure 4a shows the intensity evolution of the characteristic Raman peak of CV at 914 cm^−1^ as a function of time during the labeling/washing steps. The data points were measured at a single position on a segmented Ag line located below a nonactuated pneumatic clamp. At the beginning, a CV solution (10^−6^ m) was injected at a TFR 50 µL min^−1^ in the channel resulting in a rapid increase of the normalized Raman intensity after a short lag phase, reaching a plateau at around 200 s (Figure 4 step (i)). Afterward, the injection of DI water (at TFR 100 µL min^−1^) resulted in a decrease of the Raman signal to the initial level (Figure 4, step (ii)), thus confirming that the SERS substrates can be easily restored by a simple washing step without the need of time‐consuming and labor‐intensive procedures. Note that the actuation of the pneumatic clamp is key to keep in place physisorbed analytes like CV during the washing step.

For labeling a single Ag substrate with two different analytes, we exploited the partial actuation of pneumatic clamps. Initially, we considered a segmented Ag line labeled with CV as indicated above (Figure 3a(ii)). Injecting DI water for 15 min (at a TFR of 100 µL min^−1^), while partially actuating the pneumatic clamp (Figure 4b) allowed us to selectively wash away the analyte only from the edges of the segment. This resulted in the localized detection of CV only at the middle of the Ag line (Figure 4c). Subsequent injection of 4‐ATP (10^−6^ m) into the fluidic layer (TFR of 50 µL min^−1^ for 10 min), while maintaining the partial actuation of the pneumatic clamp, led to a sequential labeling of the Ag line with both the analytes. For example, we show in Figure 4d that with this approach a single segmented Ag line could be barcoded with a 4‐ATP/CV/4‐ATP sequence.

Remarkably, this methodology could be extended to other microengineered Ag substrates, such as an Ag film (Figure 4e; and Figure S12, Supporting Information) to form 2D SERS barcodes (Figure 4f), or to a long Ag line to generate a longer 1D barcode (Figure 4g(ii)). We observed that by varying the pressure applied to the pneumatic clamps between 0.5 and 3 bar, we could modulate the length of the region labeled with an analyte, thus increasing the SERS barcoding possibilities. For example, Figure 4g(ii) shows the Raman map of a Ag line sequentially barcoded with CV and 4‐ATP (see the Supporting Information for further details), where the distance between two analytes was modified accordingly to the actuation pressure of the pneumatic clamps (3 bar = P1 > 1 bar > P2 > P3, Figure 4g(i,ii); and Figure S13, Supporting Information). Additionally, this approach could also be extended to Ag films (Figure S14, Supporting Information). These results clearly show how the controlled pneumatic clamp actuation is crucial to keep the desired specimen at place, and thus, an important feature to enable multiple SERS detections within the same Ag substrate.

Finally, we exploited the laminar flow conditions present in our microfluidic device for the simultaneous and real‐time detection of two analytes coflowing within the fluidic layer (Figure 4h). In these experiments, two segmented Ag lines located underneath a pneumatic clamp were used as SERS active substrates (Figure 4h(i,ii)). Figure 4h(iii) shows the Raman map of the simultaneous detection of CV (10^−6^ m) and 4‐ATP (10^−6^ m) solutions injected at a TFR of 50 µL min^−1^. The data clearly demonstrate the spatial control that our method guarantees, leading different Raman probe molecules to specific detection points for a simultaneous detection along a single microfluidic channel.

The fabrication of multiple active SERS substrates inside a single microfluidic channel has been presented. This method guarantees excellent control over both the localization of SERS substrates and their aspect ratio in a straightforward manner. Additionally, we demonstrate that the SERS substrates produced in this manner can reach detection at concentrations as low as 10^−14^ m, where the diffusion of Raman probe molecules can be precisely harnessed to specific detection points, preventing cross‐contamination and endowing multiple detection capabilities. This features resulting in the generation of SERS barcodes. Additionally, an etching process with TCNQ is described that guarantees the emergence of reconfigurable, reusable, and highly flexible SERS platforms. Finally, we believe that this technology can also be used to open new avenues in other research areas that require regioselective functionalization of small‐scale metallic nanostructures, or that seek solutions to harness the growth of functional matter on surfaces. Note that a controlled growth of functional materials on surfaces is crucial to unlock their full potential into new appealing devices and technologically relevant systems.

## Experimental Section

##### Materials

The Ag salt (AgX) and reducing agent (Red) solutions (HE‐300A silver solution, HE‐300B activator solution, and HE300C reducer solution) were purchased from Peacock Laboratories (USA). TCNQ and R6G were obtained from Sigma‐Aldrich (St. Louis, USA). CV hydrate was purchased from TCI Deutschland GmbH (Germany) and 4‐ATP from Fluorochem (Glossop, UK). High performance liquid chromatography grade acetonitrile was purchased from VWR International (France). DI water was used in all the experiments.

##### Double‐Layer Microfluidic Chip Fabrication

The master molds of both the fluidic and control layers were fabricated on silicon wafers (Okmetic, Finland) coated with SU8‐2025 photoresist (Microchemicals, GmbH, Germany). Conventional soft lithography techniques were used to replicate the microchannel structures patterned on the silicon wafers.^[^ ^29^ ^]^ The height of features in both master molds was 50 µm. The top (control) layer was fabricated using a mixture of PDMS elastomer and curing agent (Sylgard 184, Dow Corning, USA) in a ratio 5:1 (weight), respectively.^[^ ^15^ ^]^ This mixture was placed under vacuum for 30 min to remove trapped gas, and then poured on top of the control layer master mold. The entire assembly was cured at 70 °C for 30 min. The fluidic layer was fabricated by spin‐coating a degassed mixture of PDMS and curing agent (20:1 in weight) onto the fluidic layer master mold. Spin‐coating consisted of two consecutive steps (500 rpm for 10 s, and then 1200 rpm for 50 s). Subsequently, the spin‐coated fluidic layer master mold was placed in an oven for 15 min at 70 °C. After dicing the control layer with a razor blade and punching the inlet holes of the pneumatic clamps with a 1 mm biopsy puncher (Miltex GmbH, Germany), the control layer was assembled on top of cured fluidic layer. To bond the two layers, the assembly was kept in an oven at 70 °C overnight. Finally, the bonded assembled layers were removed from the fluidic master mold and cut using a razor blade. Inlets and outlets holes of the fluidic layer were then punched using a 1.5 mm biopsy puncher (Miltex GmbH, Germany). The final assembly was then bonded to a glass coverslip (24 mm x 60 mm, Menzel Glasbearbeitungswerk GmbH & Co. KG, Germany) via oxygen plasma activation.^[^ ^15^ ^]^

##### Fabrication of Patterned Electrodes on a Glass Cover Slip

Interdigitated electrode patterns were fabricated on glass coverslips using a chrome mask and a lift‐off technique.^[^ ^15^ ^]^ The spacing between the electrodes was 5 µm. After spin‐coating (at 4000 rpm for 30 s) an AZ 5214 E photoresist (Clariant, GmbH) and soft baking it at 100 °C for 1 min, UV‐photolithography was performed in the hard contact mode using a Karl Süss MA/BA6 mask aligner (Süss MicroTec SE, Germany). After UV exposure, a development step was conducted to enable metallization of the structured glass coverslips with chromium and platinum (10 and 100 nm thick, respectively). Metallization was performed using an electron‐beam evaporation system (Plassys Bestek, France) prior to lift‐off using an acetone solvent.

##### Videos/Optical Images

Videos and brightfield images were taken using a Nikon Eclipse Ti microscope (Netherlands) equipped with a RETIGA R1 color camera (USA).

##### In Situ Raman Measurements

All Raman measurements were performed using an inverted Raman microscope (XploRA INV, Horiba Europe GmbH, Belgium) equipped with 532 and 785 nm lasers (see Table S1 for more details on the acquisition parameters for each experiment, Supporting Information).

##### SEM

SEM images were collected with a Zeiss Ultra 55 scanning electron microscope employing an acceleration voltage of 5.0 kV. Samples were coated before SEM imaging with 5 nm (Pt/Pd) using a Quorum Q150T‐S sputter coater.

##### XRD Analysis

Standard XRD patterns were collected with a Bruker D8 Advance powder diffractometer in Bragg‐Brentano Geometry, equipped with a MBraun PSD‐50M position sensitive detector and a curved Ge‐monochromator (Cu K*α* radiation; *λ* = 1.5418 Å). Samples were mounted on a flat glass sample plate. Patterns were collected with a step size of 0.032° and an exposure time of 1.4 s per step.

##### EDX Spectroscopy

EDX spectroscopy and elemental mapping was carried out on an FEI Quanta 200 SEM equipped with an Energy‐dispersive X‐ray analysis detector (EDAX Octane Super).

##### Electrical Conductivity Measurements

The electrical characterization of in situ grown AgTCNQ wires was performed using the glass coverslip patterned with electrodes and using a Karl Süss Prober PM8 (Süss MicroTec SE, Germany). Measurements were conducted by applying a linear voltage swept in a two‐point probe configuration.

## Conflict of Interest

The authors declare no conflict of interest.

## Supporting information

Supporting Information

Supplemental Movie 1
