# Supplementary material for: SERS Barcode Libraries: A Microfluidic Approach
Source: Adv Sci (Weinh). 2020 Apr 22;7(12):1903172. doi: 10.1002/advs.201903172 (PMC7312449; doi:10.1002/advs.201903172)
Supplement: Supplementary file 1 — Supporting Information [file ADVS-7-1903172-s001.pdf]

Copyright WILEY-VCH Verlag GmbH & Co. KGaA, 69469 Weinheim, Germany, 2020.

Supporting Information

SERS Barcode Libraries: A Microfluidic Approach

*Semih Sevim, Carlos Franco, Xiang-Zhong Chen, Alessandro Sorrenti, David Rodríguez-San-Miguel, Salvador Pané, Andrew J. deMello, Josep Puigmartí-Luis**

EXPERIMENTAL DETAILS

Electroless deposition of Ag: Two solutions were prepared; one containing a silver salt (AgX) and the other a reducing agent (Red). AgX was prepared by mixing HE300A, HE300B and deionized (DI) water (1:1:4 in volume). Red was prepared by mixing HE300C and DI water (1:5 in volume). For the electroless deposition of two segmented Ag lines, the concentrations of both the AgX and Red solutions were optimized. Specifically, the previously prepared AgX and Red solutions were diluted 10 times. This dilution was critical in allowing the readjustment of flows before electroless deposition of Ag at the interface of the two co-flowing (reagent-laden) streams.[14]

Segmented Ag lines: Initially, a continuous Ag line was patterned by injecting AgX and Red solutions (at equal flow rates) though inlet 1 and inlet 2, respectively (Figure 1a). The width of the in-flow patterned Ag lines could be controlled by varying the total flow rates (TFR), ranging from 20 µl/min to 100 µl/min (Figure S3). After formation of a continuous Ag line in the fluidic layer, a washing step with DI water (50 µl/min for 3 minutes) was conducted to remove unreacted solutions present inside the fluidic layer. This is followed by another washing step using acetonitrile (50 µl/min for 3 minutes) to exchange the solvent before introduction of a saturated TCNQ solution in acetonitrile (50 µl/min). Injection of a saturated TCNQ solution enabled the etching of the Ag line from the glass substrate, with pressurization of the pneumatic clamps (using nitrogen gas at 3 bar) preventing the etching of

Ag. Next, a washing step with acetonitrile (50 µl/min for 3 minutes) was used to remove the excess of TCNQ from the microfluidic channel. As a result, segmented Ag lines were patterned on the glass substrate only where pneumatic clamps were actuated.

Two segmented Ag lines: Diluted AgX and Red solutions were used in this experiment. In a typical synthesis, an Ag line was fabricated by injecting AgX and Red solutions at flow rate 30 µl/min and 50 µl/min, respectively. Subsequently, a second Ag line was generated by co-flowing AgX and Red solutions at a flow rate of 50 µl/min and 30 µl/min, respectively. The change in flow rate enabled precise positioning of the reaction interface within the main microfluidic channel. This is important, since the interface of the co-flowing AgX and Red flows defines the where electroless deposition of Ag occurs. Accordingly, the controlled positioning of the interface inside the main microfluidic channel allows the generation of multiple Ag lines. To obtain two segmented Ag lines underneath a clamp an etching step with TCNQ was performed as described above.

Detection and statistical analysis: In this experiment, an aqueous solution of CV of different concentration (ranging between 10^{-14} M and 10^{-6} M) was injected over a period of 4 minutes at a constant TFR of 50 µl/min and Raman spectra were recorded just after stopping the flow at 31 selected points along an Ag line. The intensity of a characteristic Raman peak for CV (i.e. the peak at 1173 cm^{-1}) was measured at the different points for each CV concentration. We set a threshold, and considered a successful analyte detection when the height of the characteristic Raman peak exceeded 200 counts (that corresponds to a recognizable Raman spectrum for CV). This process was repeated 3 times for each concentration, leading to an averaged value that was then represented in the graph as detection event percentage (Figure 2g). The error bars presented in Figure 2g represent the root mean square error (RMSE) obtained from the three repetitions.

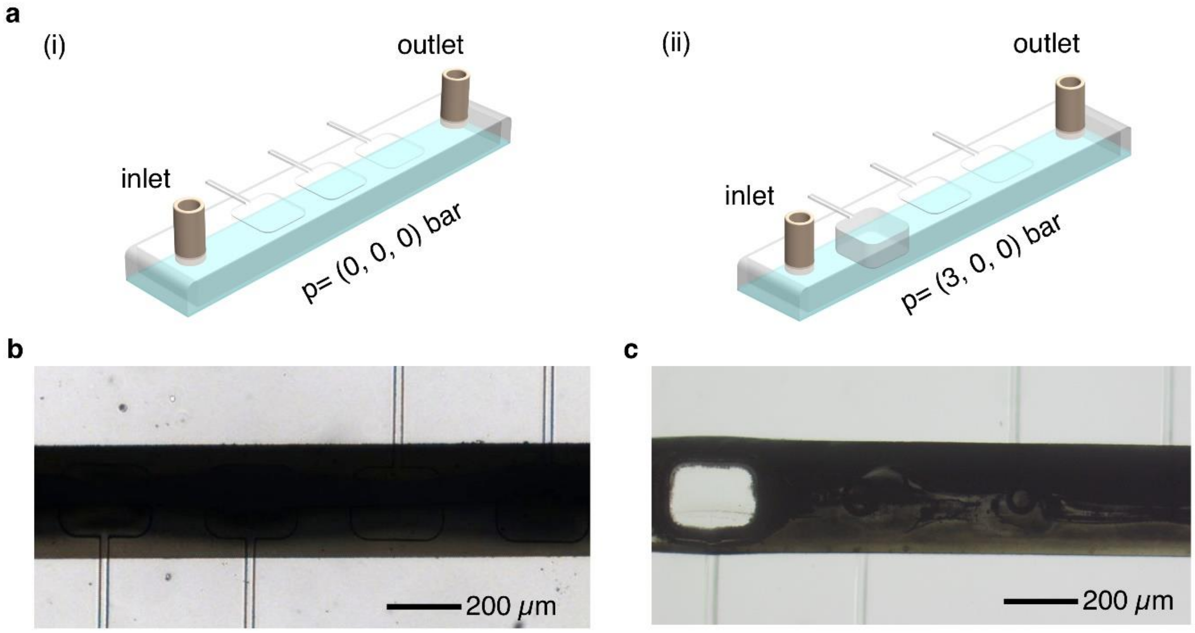

Figure S1. (a) Schematic representations of a double layer microfluidic device that comprises a single inlet and outlet. (i) the pneumatic clamps are not actuated (0 bar), (ii) the first clamp is actuated at 3 bar. (b) Brightfield image of an electroless deposited Ag film within the fluidic layer. (c) Brightfield image of a microengineered Ag film containing an empty patch. Here, the first pneumatic clamp was actuated during the electroless deposition of Ag, as shown in (ii).

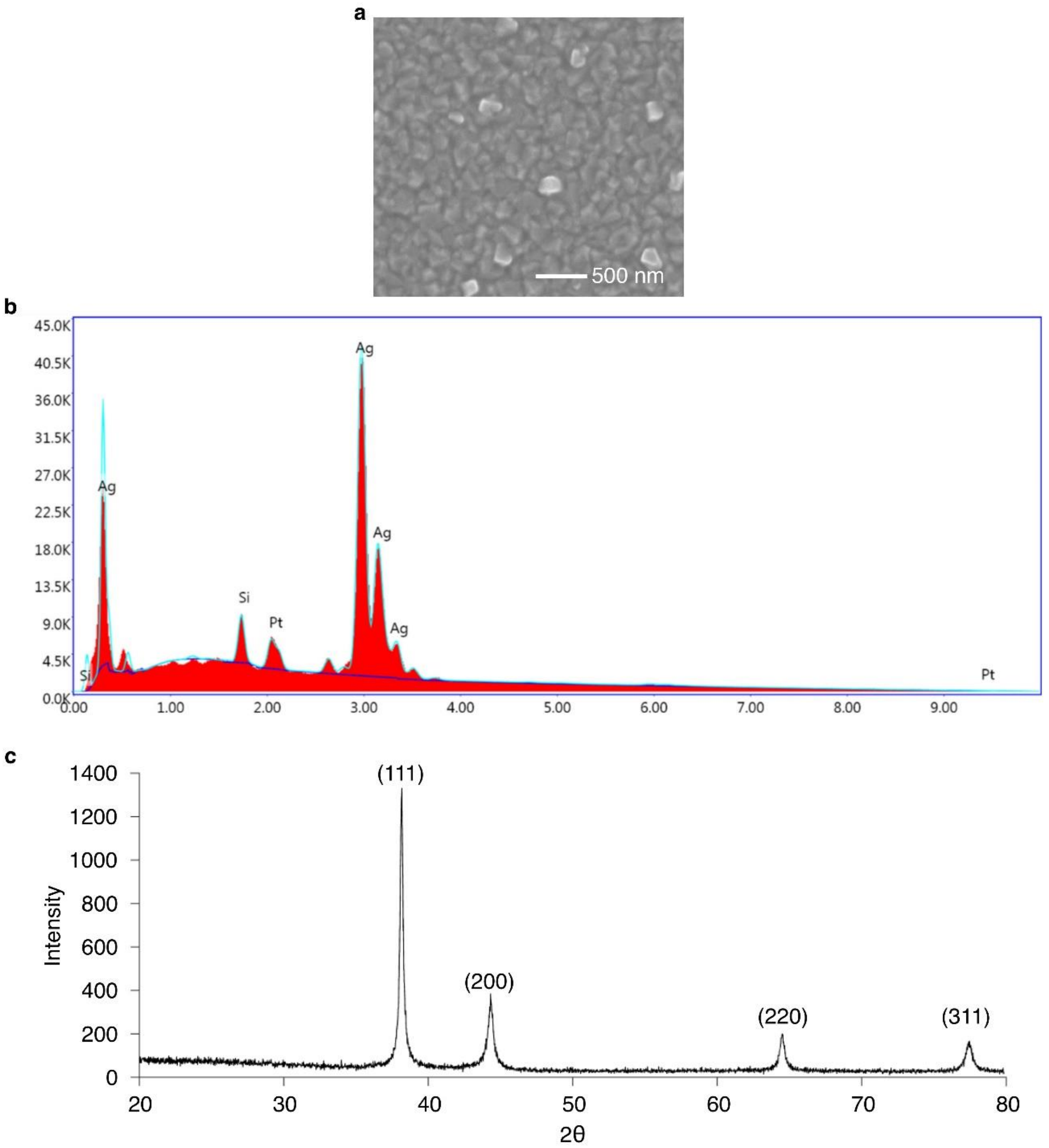

Figure S2. (a) SEM image and (b) EDX spectrum of an electroless deposited Ag film. (c) XRD spectrum of the same Ag film. The diffraction peaks annotated correspond to a face-centered cubic (fcc) Ag structure.[16]

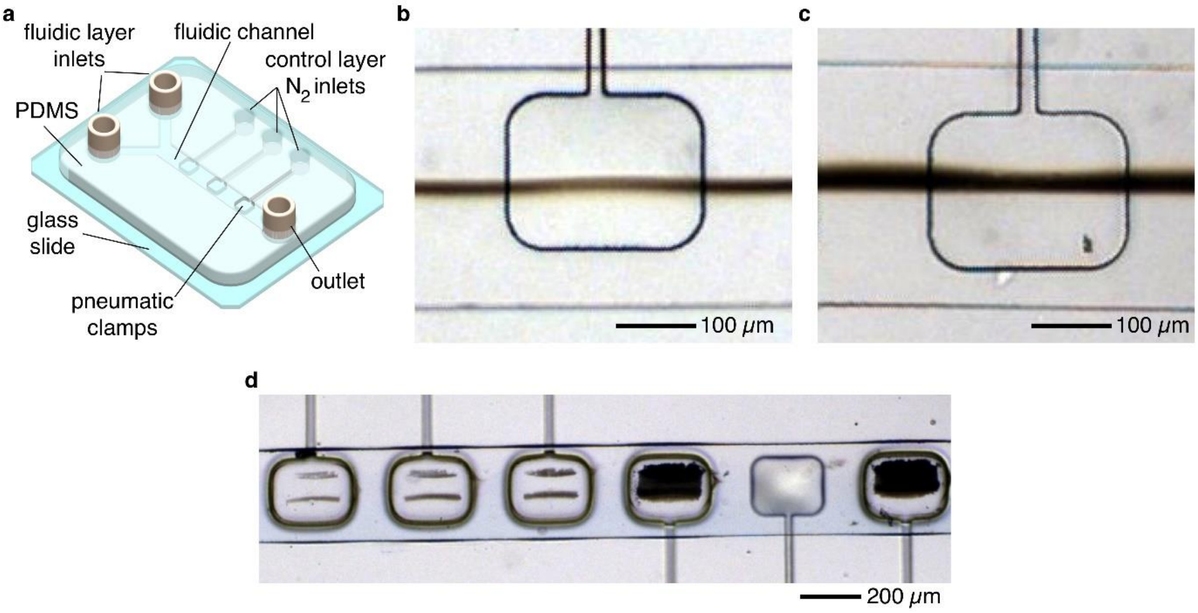

Figure S3. (a) Schematic illustration of a double layer microfluidic device containing a Y-shape channel pattern. (b) and (c) Images of *in-situ* patterned Ag lines with different width. In (b) the width of the Ag line is ca. 12 µm and in (c) ca. 36 µm. (d) Image showing the results of adjacent synthesis of different micro-engineered Ag substrates underneath a sequence of pneumatic clamps and within a single microfluidic channel.

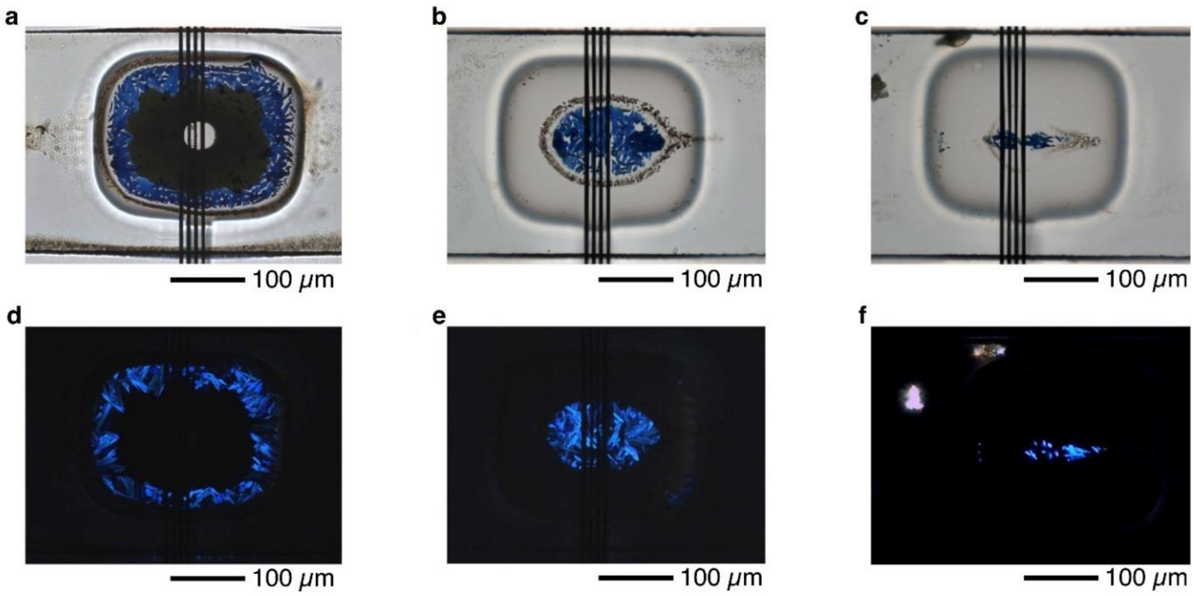

Figure S4. (a-c) Brightfield and (d-f) cross-polarized images of AgTCNQ wires. In (a), (b), (d) and (e) the growth of the AgTCNQ wires is directed towards the center of the Ag film previously deposited, whereas in (c) and (f) AgTCNQ wires are aligned in the direction of the flow. In (c), an Ag line was used as a template to grow AgTCNQ wires.

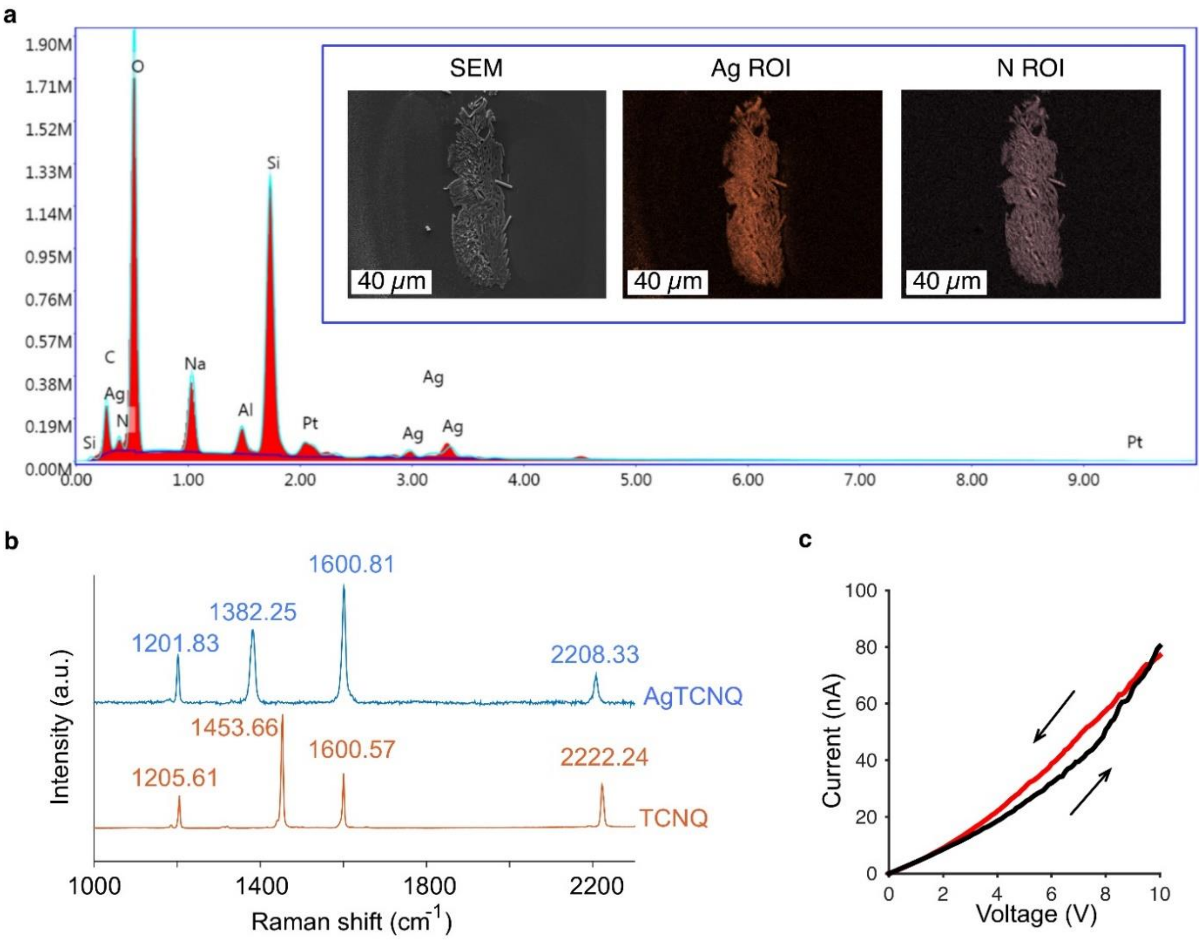

Figure S5. (a) EDX spectrum of AgTCNQ wires. The inset shows a SEM image of AgTCNQ wires together with corresponding EDX maps of Ag and N. (b) Raman spectra of AgTCNQ wires (blue) and TCNQ (orange), showing characteristic Raman peaks of both compounds. (c) I-V curve measured from AgTCNQ wires.

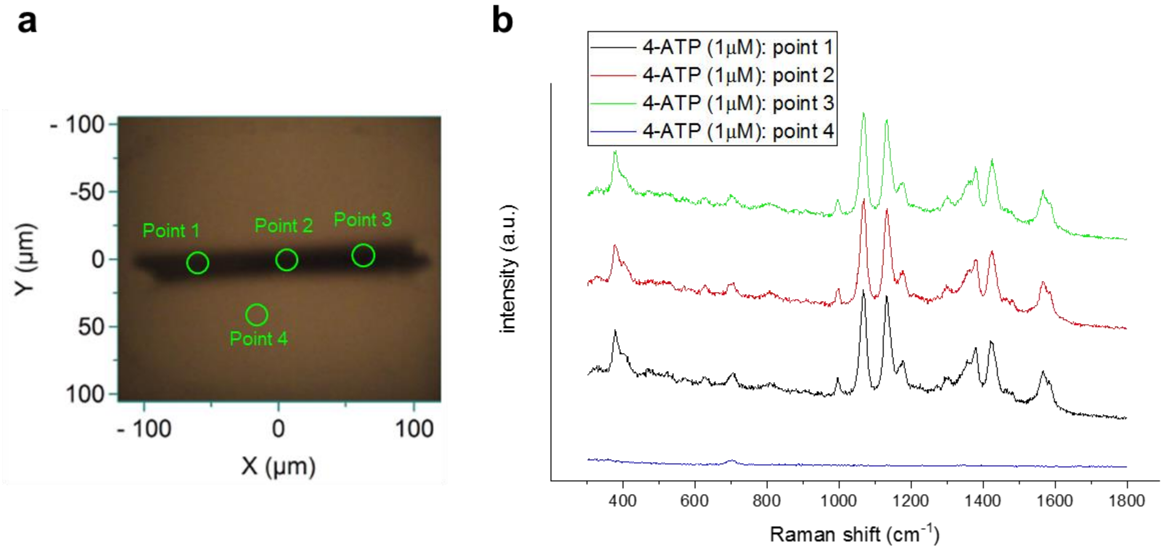

Figure S6. (a) Optical microscope image of the Ag line used for 4-ATP detection. The circles in the image indicate the positions were the Raman measurements were performed. (b) Raman spectra acquired on the positions indicated in (a). The blue spectra was measured outside the Ag line, i.e. on the glass substrate. The concentration of 4-ATP used in this experiment was 10^{-6} M.

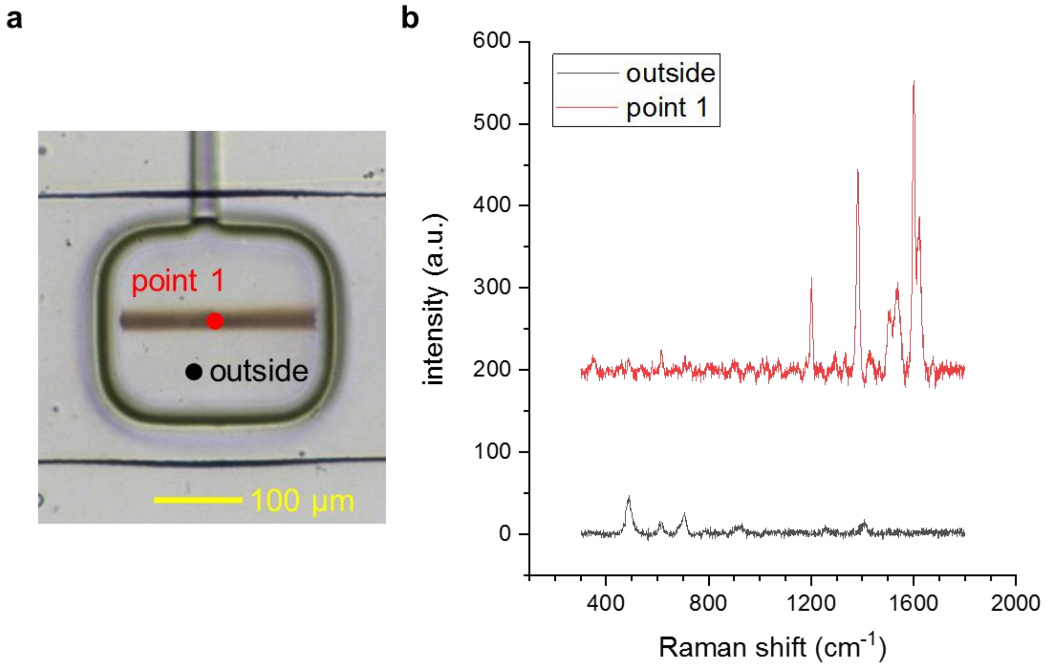

Figure S7. (a) Optical microscope image of the Ag line used for BSA (10^{-4} M) detection. The BSA solution was pumped through microchannel with a flow rate of 50 µl/min during 15 minutes. The points in the image indicate the representative positions were the Raman measurements were performed. (b) Raman spectra acquired directly after stopping the flow on the positions indicated in (a). The black spectra was measured outside the Ag line, i.e. on the glass substrate whereas the red spectra acquired on top of the Ag line clearly showing the characteristic Raman peaks of BSA.

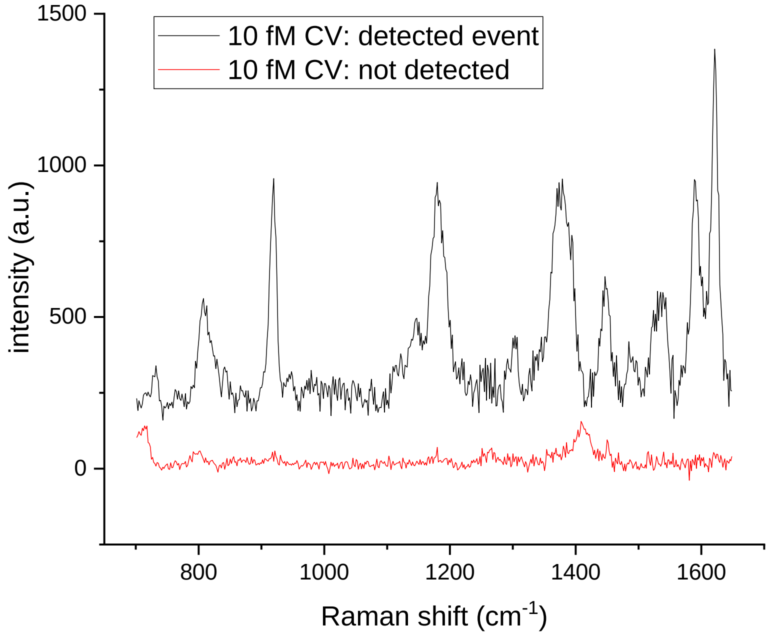

Figure S8. Representative Raman spectra obtained during the detection event percentage experiment shown in Figure 2g. A successful analyte detection event is presented in black and an unsuccessful one in red. The concentration used in this measurement was 10^{-14} M.

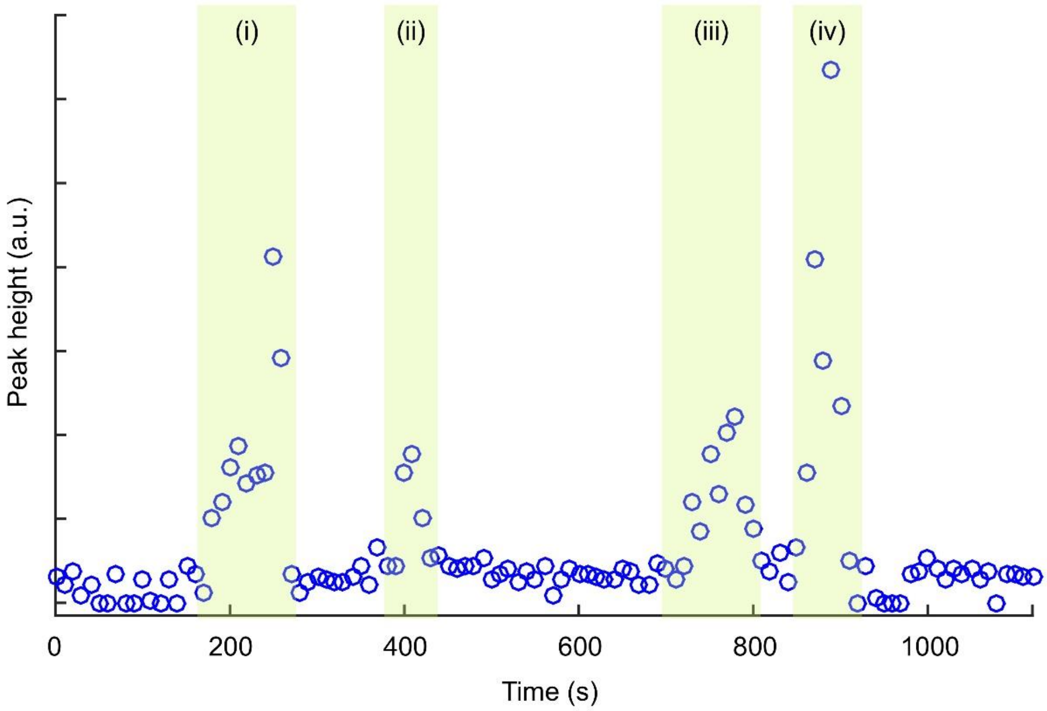

Figure S9. Variation of Raman peak intensity (1173 cm^{-1}) at as a function of time (taken at a single point on a segmented Ag line). During the experiment, a CV solution (10^{-9} M) was injected into the fluidic layer at a constant TFR of 50 µl/min. Regions (i-iv) represent the random time-lapse where the height of a characteristic Raman peak for CV increased. These stochastic detection events suggest that few/single molecules are detected under these conditions.

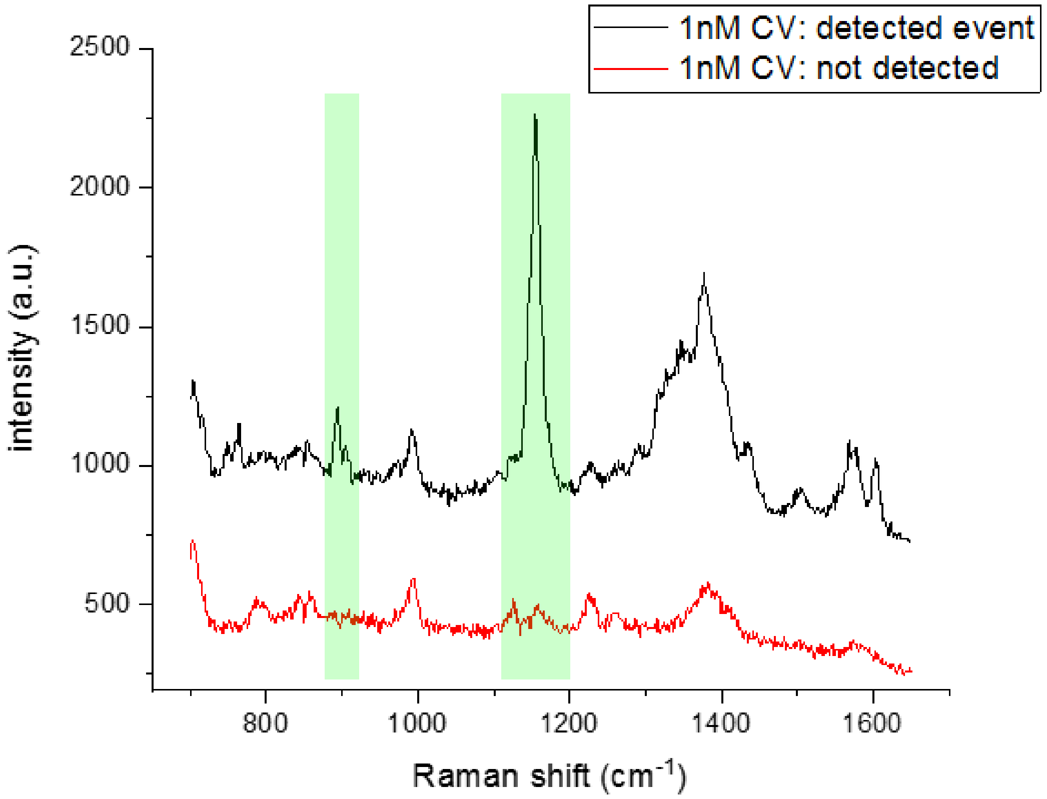

Figure S10. Representative Raman spectra obtained during the continuous flow detection of CV (see Figure S9). While the red spectra indicates an unsuccessful detection event (the characteristic peaks of CV located 914 cm^{-1} and 1173 cm^{-1} are clearly missing in the red spectra), the black spectra denotes a successful one. In the graph, green marks indicate the characteristic peaks used to confirm CV detection. As indicated in Figure S9, the CV concentration used for this experiment was 10^{-9} M.

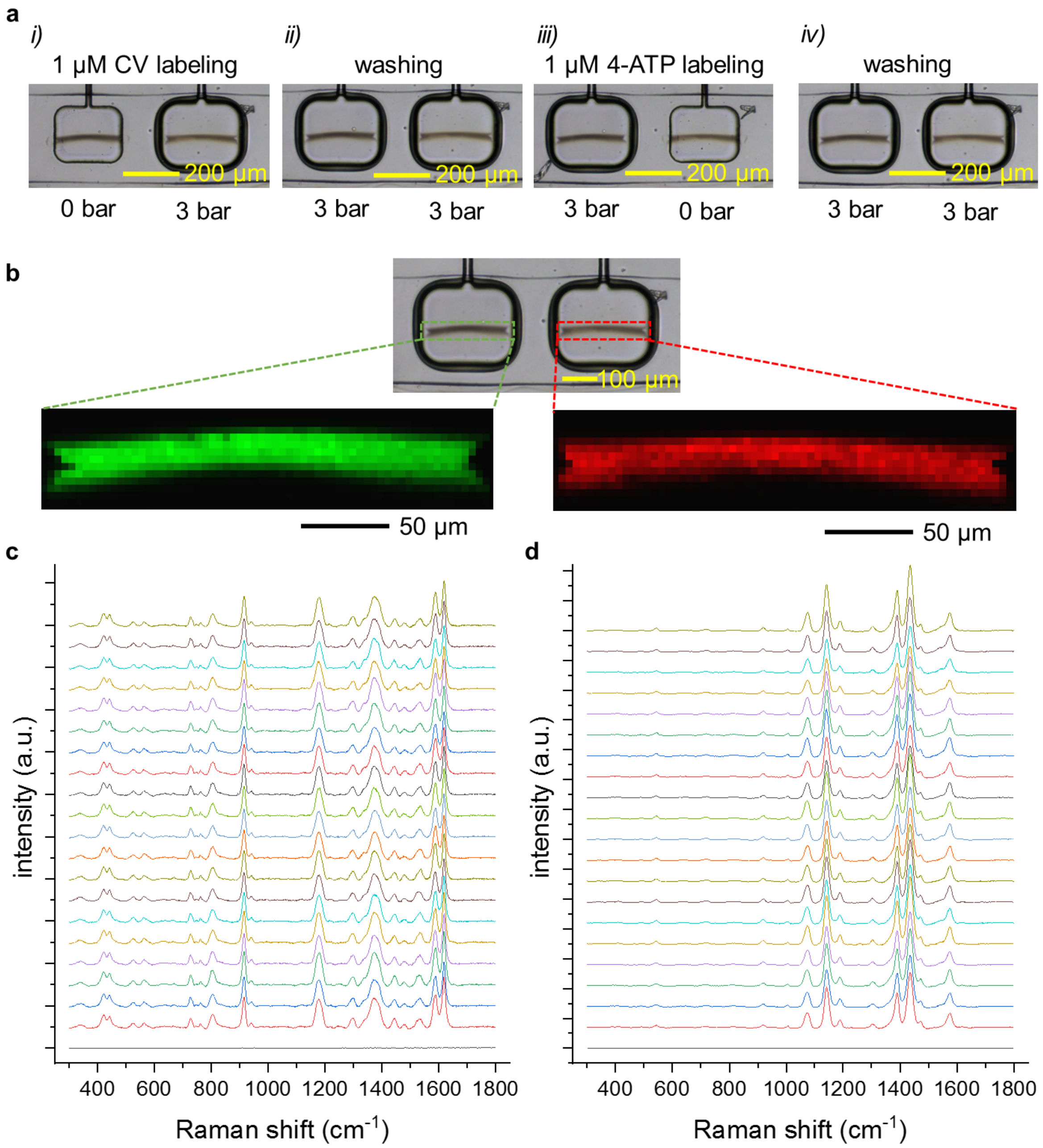

Figure S11. (a) From (i) to (iv) microscope images showing the sequential steps followed to achieve the simultaneous detection of two model Raman probe molecules on segmented Ag lines located within a single microfluidic channel. (b) Microscope image of the two adjacent segmented Ag lines used in the experiment together with false color Raman map (4 µm x 4 µm resolution) of the two Raman probe molecules on each segmented Ag line. CV is represented by green and 4-ATP by red. (c-d) 20 representative SERS spectra obtained on each Ag line and one representative spectra obtained from outside of the Ag line during the mapping shown in (b) respectively indicating pure CV(c) and 4-ATP(d) characteristic Raman peaks.

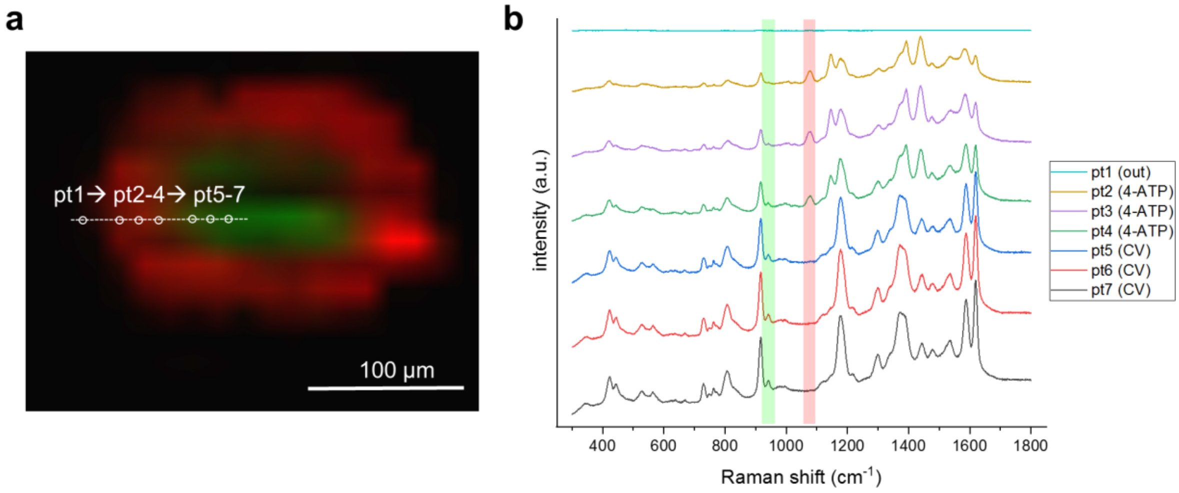

Figure S12. (a) Color coded Raman map from Figure 4f showing the 2D SERS barcode generated with our approach. The Raman spectra presented in (b) correspond to the seven points (pt) marked in (a). The points over the dashed line indicate where the Raman spectra presented in (b) are collected from the whole 2D Raman map. Clearly, pt1 was measured outside the Ag film (light blue spectra). As it can be clearly seen in b, the light blue spectra does not have characteristic peaks of CV or 4-ATP. From pt2 to pt4 the Raman spectra were collected on the 4-ATP labeled region, and from pt5 to pt7 in the CV labeled area. In (b), green and red rectangles highlight the selected peaks used to locate CV and 4-ATP on the Ag film, respectively, and to create the color coded Raman map. This experiment clearly shows that there is no cross-contamination between analytes with our approach.

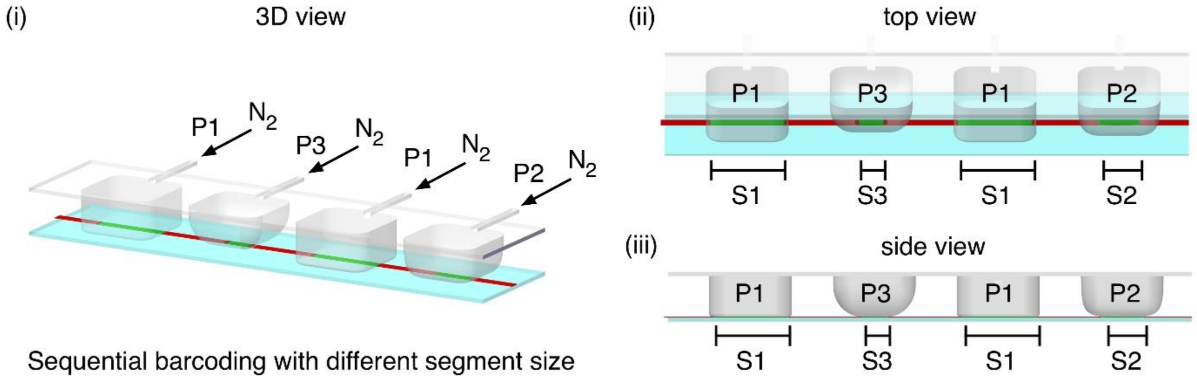

Figure S13. Schematic illustrations showing a sequentially barcoded Ag line. (i) 3D view, (ii) Top view and (iii) Side view of a double-layer microfluidic device containing a barcoded Ag line. The barcode is generated with two different analytes, represented in red and green. As shown in (ii) and (iii), varying the pressure of pneumatic clamps (3 bar = P1 > 1bar > P2 > P3) leads to barcodes of variable length.

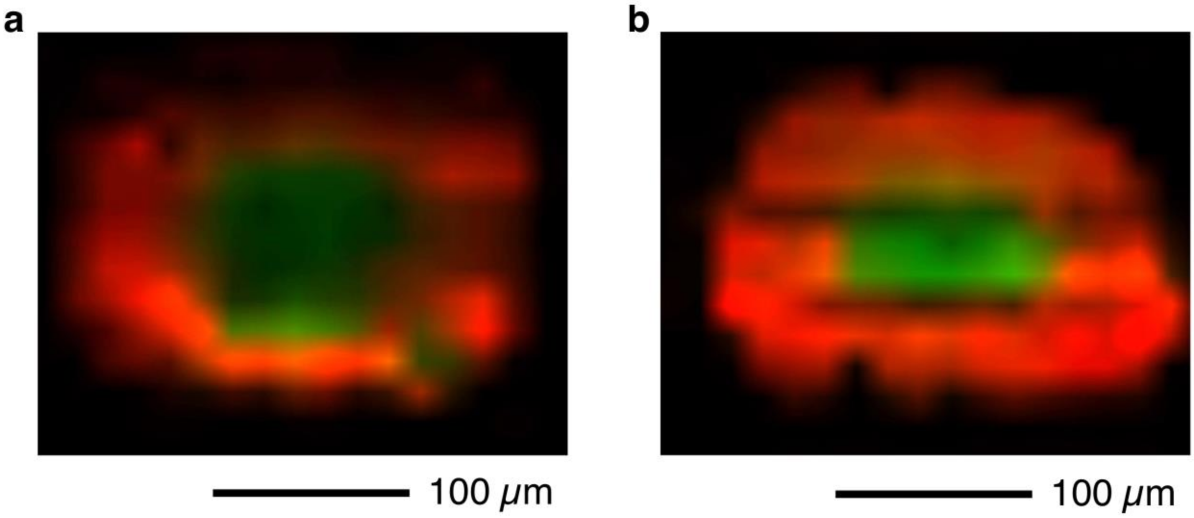

Figure S14. (a) and (b) Color-coded Raman maps from two different Ag films partially labeled with CV (10^{-6} M, green) and 4-ATP (10^{-6} M, red). It should be noted that in (a) the CV labeled region is larger than in (b). Varying the size of the CV labeled region can be easily accomplished by controlling the nitrogen pressure applied to the pneumatic clamp. In (a), the clamp membrane is deflected more strongly towards the glass substrate than in (b).

Table S1. Raman parameters used in the acquisition of different data sets presented in the main text and Supporting information.

Figure Number	Objective	Laser	Power	Acquisition [sec.]	Accumulation
Figure 2f	10x	532 nm	0.1 %	2 sec	10
Figure 2g	10x	532 nm	1.0 %	5 sec	1
Figure 3c	40x	785 nm	1.0 %	5 sec	2
Figure 4a	10x	532 nm	1.0 %	1 sec	1
Figure 4c	40x	785 nm	1.0 %	5 sec	2
Figure 4d	40x	785 nm	1.0 %	5 sec	2
Figure 4f	10x	532 nm	1.0 %	5 sec	1
Figure 4g	40x	532 nm	1.0 %	5 sec	2
Figure 4i	10x	532 nm	1.0 %	5 sec	2
Figure S6	10x	785 nm	1.0 %	10 sec	2
Figure S7	40x	532 nm	1.0 %	10 sec	10
Figure S8	10x	532 nm	1.0 %	5 sec	1
Figure S9	10x	532 nm	1.0 %	1 sec	1
Figure S10	10x	532 nm	1.0 %	1 sec	1
Figure S11	40x	532 nm	1.0 %	1.5 sec	3
Figure S12	10x	532 nm	1.0 %	5 sec	1
Figure S14	10x	532 nm	1.0 %	5 sec	1
